# Comparison of violence and aggressions suffered by health personnel during the COVID-19 pandemic in Argentina and the rest of Latin America

**DOI:** 10.17843/rpmesp.2023.402.12646

**Published:** 2023-06-30

**Authors:** Sebastián Garcia-Zamora, Pablo A. Iomini, Laura Pulido, Andrés F. Miranda-Arboleda, Pilar Lopez-Santi, Lucrecia M. Burgos, Gonzalo E. Perez, Mauricio Priotti, Darío E. García, Melisa Antoniolli, Gabriel Musso, Ezequiel J. Zaidel, Álvaro Sosa-Liprandi, Mildren A. Del-Sueldo, Ricardo Lopez-Santi, Gustavo Vazquez, Adrián Baranchuk

**Affiliations:** 1 Servicio de Cardiología, Sanatorio Delta, Rosario, Argentina. Servicio de Cardiología Sanatorio Delta Rosario Argentina; 2 Facultad de Medicina, Universidad de Buenos Aires (UBA) - UDH Hospital Dr. Prof. Alejandro Posadas, Buenos Aires, Argentina. Universidad de Buenos Aires Facultad de Medicina Universidad de Buenos Aires (UBA) UDH Hospital Dr. Prof. Alejandro Posadas Buenos Aires Argentina; 3 Pneumonology Service, Sanatorio Centro, Rosario, Argentina. Pneumonology Service Sanatorio Centro Rosario Argentina; 4 Cardiology Division, Queen’s University, Kingston, Ontario, Canada. Cardiology Division Queen’s University Kingston Ontario Canada; 5 Cardiology Division, Hospital Italiano de La Plata, La Plata, Argentina. Cardiology Division Hospital Italiano de La Plata La Plata Argentina; 6 Instituto Cardiovascular de Buenos Aires, Argentina. Instituto Cardiovascular de Buenos Aires Argentina; 7 Cardiology Division, Clínica Olivos, Buenos Aires, Argentina. Cardiology Division Clínica Olivos Buenos Aires Argentina; 8 Latin American Federation of Emergency Medicine, Ciudad de Mexico, Mexico. Latin American Federation of Emergency Medicine Ciudad de Mexico Mexico; 9 Cardiology Service, Sanatorio Finochietto, Buenos Aires, Argentina. Cardiology Service Sanatorio Finochietto Buenos Aires Argentina; 10 Intensive Care Service, Sanatorio Parque, Rosario, Argentina. Intensive Care Service Sanatorio Parque Rosario Argentina; 11 Cardiology Department, Sanatorio Güemes, CABA, Argentina. Cardiology Department Sanatorio Güemes CABA Argentina; 12 Certus-Clínica de Especialidades Foundation, Cordoba, Argentina. Certus-Clínica de Especialidades Foundation Cordoba Argentina; 13 Department of Psychiatry, Queen’s University, Kingston, Ontario, Canada. Department of Psychiatry Queen’s University Kingston Ontario Canada

**Keywords:** Violence, COVID-19, Pandemics, Aggression, Latin America, Health Personnel

## Abstract

**Objectives.:**

To explore the frequency and impact of violence against healthcare workers in Argentina and to compare it with the rest of their Latin American peers during the COVID-19 pandemic.

**Materials and methods.:**

A cross-sectional study was conducted by applying an electronic survey on Latin American medical and non-medical personnel who carried out health care tasks since March 2020. We used Poisson regression to estimate crude (PR) and adjusted (aPR) Prevalence Ratios with their respective 95% confidence intervals.

**Results.:**

A total of 3544 participants from 19 countries answered the survey; 1992 (56.0%) resided in Argentina. Of these, 62.9% experienced at least one act of violence; 97.7% reported verbal violence and 11.8% physical violence. Of those who were assaulted, 41.5% experienced violence at least once a week. Health personnel from Argentina experienced violence more frequently than those from other countries (62.9% vs. 54.6%, p<0.001), and these events were more frequent and stressful (p<0.05). In addition, Argentinean health personnel reported having considered changing their healthcare tasks and/or desired to leave their profession more frequently (p<0.001). In the Poisson regression, we found that participants from Argentina had a higher prevalence of violence than health workers from the region (14.6%; p<0.001).

**Conclusions.:**

There was a high prevalence of violence against health personnel in Argentina during the COVID-19 pandemic. These events had a strong negative impact on those who suffered them. Our data suggest that violence against health personnel may have been more frequent in Argentina than in other regions of the continent.

## INTRODUCTION

The COVID-19 pandemic has caused multiple negative repercussions on both individuals and healthcare systems ^1^^-^^3^. Social distancing, economic and labor problems, and changes in the habits and routines of individuals during this period have been linked to increased stress, anxiety, and depression in different societies ^2^. At the same time, health personnel have been overloaded with work and have had to modify their methods and alter their routines to help them cope with this complex situation ^4^. In this unfavorable scenario, one problem has arisen: aggression against health personnel ^5^^-^^7^.

Violence against health personnel has been a global phenomenon of concern for decades ^8^^-^^11^. In 2002, the World Health Organization (WHO) and partner organizations outlined guidelines to address this problem. They defined workplace violence as “incidents in which staff are abused, threatened or assaulted in circumstances related to their work [... ] which involve an explicit or implicit challenge to their safety, well-being or health” ^12^.

Despite the above, little is known about what has happened in Latin America or Argentina after the onset of the COVID-19 pandemic. Therefore, the Inter-American Society of Cardiology (SIAC) conducted a survey to explore the frequency and characteristics of violence against healthcare workers in Latin America during the COVID-19 pandemic ^13^. In this survey, more than 50% of the participants reported having experienced some episode of workplace violence during the pandemic. Despite the high figure, this does not seem to be unique to Latin America; similar studies in China and the United States have reported figures of over 65%, even reaching 80% of the participants in some studies ^6^^,^^7^. The impact of regional variations on the frequency of violent events in the SIAC survey is not clear. In the present sub-study, we aimed to explore the frequency of violent events, and the characteristics and impact of these events on health care workers in Argentina, as the country with the largest number of participants in the study, and to compare it with the rest of Latin American countries in the context of the COVID-19 pandemic.

**KEY MESSAGES**

Motivation for the study. The COVID-19 pandemic has caused profound repercussions at different socio-environmental levels. Its impact on violence against healthcare team workers in Argentina has not been well documented.

Main findings. The present study evidenced high rates of aggression, particularly verbal aggression. In addition, almost half of the participants reported having suffered these events on a weekly basis. All participants who experienced violence reported having experienced post-event symptoms, and up to one-third reported having considered changing their profession after these acts.

Implications. It is imperative to take action to prevent acts of violence against health personnel, or to mitigate its impact on the victims.

## MATERIALS AND METHODS

### Design and study population

The development and implementation of the project have been published previously ^13^. In brief, a cross-sectional study was carried out using a self-administered electronic survey, which was designed specifically for this study. The survey was administered between January 11 and February 28, 2022, and all medical and non-medical health personnel in Latin America were invited to participate. Participation in the survey was voluntary and anonymous, and this information was provided to participants at the beginning of the questionnaire. Health personnel were defined as all members of the system, including physicians, nurses, kinesiologists, biochemists, technicians in different areas (diagnostic imaging, cardiology practices, laboratory, among the main ones), and administrative personnel who had contact with patients or family members. The physicians were not restricted by specialty or work, place of residence, age, or other variables. We excluded those individuals who had not performed healthcare activities since the beginning of the pandemic in the region, setting March 2020 as the time limit for this purpose. We did not calculate the sample size due to the lack of reliable data on the number of health professionals in Latin America.

The questionnaire was distributed electronically through invitations sent to the e-mail addresses of SIAC members, both by SIAC and its member scientific societies. In addition, while the survey was being applied, invitations were also sent through SIAC’s social media platforms (Twitter, WhatsApp and Instagram).

### Questionnaire

The survey was designed in Google Forms (Mountain View, CA), following the recommendations of the Consensus for Reporting Survey Studies (CROSS) proposed by the Quality Enhancement and Transparency in Health Research (EQUATOR) Network ^14^. Forty-nine questions divided into five sections were included; with it we collected data regarding demographic characteristics of the participants, their training and experience, degree of association with patients with active COVID-19 infection, and questions related to possible experiences of violence or aggression during the period analyzed (Supplementary Material).

Due to the dynamic nature of the events and repercussions of the COVID-19 pandemic, and given that the potential participants were highly educated individuals, we decided not to conduct a pilot test.

### Variables and measurements

The definitions of the different types of violence were based on WHO guidelines ^(^^12^^,^^13^. Thus, violence was considered to be any type of incident related to patients, patients’ relatives or any other individual who is not part of the health institution, where the health personnel felt that their safety was at risk. The definition of other types of violence, as well as the psychological consequences of these events (reviviscence, withdrawal symptoms, hypervigilance and reactivity, and cognitive symptoms or mood alterations) are detailed in the Supplementary Material.

A linear Likert-type scale was used to assess the level of stress perceived by the participants who experienced violent events. Participants who had suffered an act of violence were asked to indicate how stressful the experience had been for them, using a scale from 1 to 10. On this scale, assigning a score of 1 meant that the event had not been stressful, while a score of 10 was equivalent to the most stressful work event they had experienced (Supplementary Material).

### Statistical analysis

Continuous variables were expressed as mean and standard deviation or median and interquartile range, according to their distribution. The normality of each variable was evaluated using graphic tools (histograms and normal probability plots) and the Shapiro-Wilk test. Categorical variables were expressed as absolute values and percentages. Student’s t-test was used for comparisons between groups of continuous variables that were normally distributed. Comparisons between proportions were performed using the chi-square test or Fisher’s exact test according to the frequency of expected values. Missing data were detected in three survey items: sex of participants (0.3%), country of residence (0.2%), and year of receipt (3.3%); however, no strategy was applied to compensate for this issue.

Subsequently, a regression model was constructed to explore whether participants from Argentina experienced episodes of violence with a different frequency than health personnel from other Latin American countries. A Poisson regression was used in order to estimate the prevalence ratio of violence events among participants. First, the prevalence ratio of suffering some type of violence was calculated for all the variables collected in the survey (crude analysis). Then, we proceeded to construct a manually adjusted model, including only the variables that obtained a value of p<0.20 in the crude model. In the final adjusted model, we only retained only the variables that showed statistical significance (p<0.05). Finally, the presence of collinearity in the variables of the adjusted model was explored by calculating the variance inflation factor (VIF). A VIF of 1 indicated the absence of collinearity, while a value greater than 10 was considered as high collinearity. Statistical significance was established as a value of p<0.05 in two-tailed tests for all statistical tests. All analyses were carried out with Stata version 13.0 (StataCorp, College Station, TX, USA).

### Ethical Aspects

The research protocol was approved by the SIAC Ethics Committee, and under no circumstances was the integrity or information of the participants put at risk. With the aim of guaranteeing anonymity and promoting the veracity of the responses, no identifying information was requested from the participants, nor was written consent required. Thus, we considered that those who responded to the survey tacitly gave their informed consent to participate in the study ^15^. To ensure that responses were not duplicated, the questionnaire was programmed to collect participants’ e-mail addresses on a mandatory basis. Only the principal investigator had access to these data, and was responsible for anonymizing them, thus guaranteeing the confidentiality of the participants.

## RESULTS

A total of 3544 participants from 19 countries responded to the survey; of these, 56.2% (n=1992) resided in Argentina. Sixty-three percent of the participants from Argentina were women, and the mean age of the participants was 42.6 ± 10.7 years. Of those from Argentina, 68.4% were physicians, 19.9% were nurses, 3.2% were kinesiologists, 3.0% were secretaries or administrators, and the remaining 5.5% had other functions within the healthcare team. Of the physicians, 91.5% stated that they were specialists: 34.4% were cardiologists, 10.5% were intensivists or emergency specialists, 11.4% had a surgical specialty, 9.4% were pediatricians or had a related subspecialty. Of the participants, 75.1% reported regularly worked with patients with COVID-19. Table 1 compares the baseline characteristics of the participants from Argentina and those from the rest of the continent.

**Table 1:** Baseline characteristics of participants from Argentina compared with other countries in the Americas.

Characteristic	Argentina (n=1992)	Rest of Latin America (n=1552)	p-value ^a^
	n (%)	n (%)	
Sex (n=3532)			
Women	1251 (63.0)	816 (52.8)	<0.001
Men	734 (37.0)	731 (47.2)	
Age (years)^b^	42.6 ± 10.7	41.1 ± 11.3	<0.001^c^
Health personnel (n=3544)			
Physicians	1363 (68.4)	1147 (73.9)	<0.001
Nurses	397 (19.9)	170 (11.0)	<0.001
Kinesiologists	63 (3.2)	56 (3.6)	0.465
Administrative	59 (3.0)	41 (2.6)	0.568
Other ^d^	110 (5.5)	138 (8.9)	<0.001
Specialists (n=2498)			
Yes	1243 (91.5)	882 (77.4)	<0.001
No	116 (8.5)	257 (22.6)	
Experience (n=3364)			
Up to 10 years	728 (38.2)	717 (49.1)	<0.001
From 11 to 15 years	383 (20.1)	210 (14.4)	
Over 15 years	793 (41.7)	533 (36.5)	
Field of work (n=3525)			
Public	677 (34.1)	610 (39.6)	0.001
Private	614 (31.0)	405 (26.3)	
Public and private	693 (34.9)	526 (34.1)	
Worked with patients with COVID-19 (n=3544)			
Yes	1496 (75.1)	1151 (74.2)	0.809
No	496 (24.9)	401 (25.8)

a calculated using the chi-square test; ^b^ Mean ± standard deviation; ^c^ calculated using Student’s t-test; ^d^ technicians in different areas, biochemists, nutritionists, and speech therapists.

Among the Argentine population, 62.9% reported having suffered at least one act of violence after the onset of the COVID-19 pandemic. A total of 97.7% suffered verbal violence, 11.8% physical violence and 39.4% other types of violence. The victims rated the stress level of these events with an average score of 8.4 ± 1.7 points. Although there were no differences in the frequency of aggressions between Argentina and other countries according to sex (97.7% in women vs. 97.6% in men, p=0.945), women experienced these events in a slightly more stressful way (with an average score of 8.6 ±1.5 vs. 8.2 ±1.9 in men, p<0.001). Of those assaulted, 12.9% stated that they suffered some type of violence on a daily basis; 28.6% experienced this approximately once a week, and 36.8% expressed suffering it a few times a month. The aggressions were mainly carried out by patients and their relatives (34.7%), or only by relatives (31.0%) (Table 2).

**Table 2:** Differences in patterns of violence between health personnel in Argentina and those in other regions of Latin America.

Characteristics	Argentina (n=1992)	Other regions (n=1552)	p-value ^a^
	n (%)	n (%)	
Overall violence			
Yes	1253 (62.9)	847 (54.6)	<0.001
No	739 (37.1)	705 (45.4)	
Verbal violence			
Yes	1224 (97.7)	816 (96.3)	0.069
No	29 (2.3)	31 (3.7)	
Physical violence			
Yes	148 (11.8)	85 (10.0)	0.204
No	1105 (88.2)	762 (90.0)	
Other types of violence			
Yes	493 (39.4)	259 (30.6)	<0.001
No	760 (60.6)	588 (69.4)	
Time of occurrence of violent events			
Weekly ^b^	520 (41.5)	309 (36.5)	0.021
Less than once a week	733 (58.5)	538 (63.5)	
Perceived stress score ^c,d^	8.4 ±1.7	7.8 ±2.1	<0.001 ^d^
Required psychological care			
Yes	164 (13.1)	96 (11.3)	0.231
No	1089 (86.9)	751 (88.7)	
Evidenced violence against other members of the health care team.			
Yes	1548 (77.7)	1107 (71.3)	<0.001
No	444 (22.3)	445 (28.7)	

a calculated using the chi-square test; ^b^ participants reported having suffered episodes of violence at least once a week; ^c^ mean ± standard deviation; ^d^ based on a score from 1 to 10, where 1 is minimally stressful and 10 is the most stressful event they have experienced; ^e^ calculated using Student's t-test.

**Figure 1:**
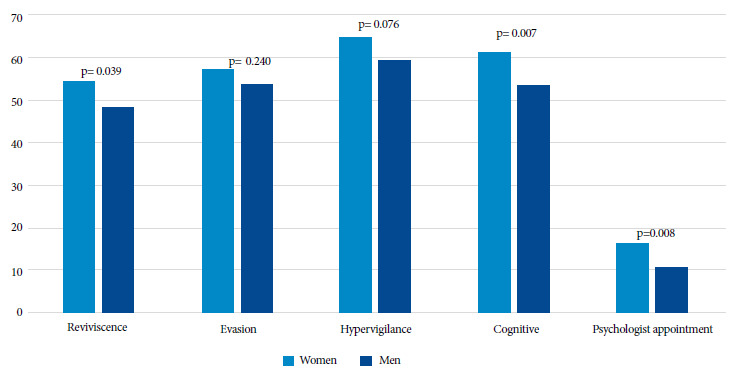
Symptoms experienced by participants in Argentina who experienced at least one episode of aggression or abuse, according to sex.

All participants from Argentina who experienced violence reported having experienced post-event symptoms, with hypervigilance being the most frequent (63.2%). Flashbacks and cognitive symptoms were more frequent among women, as well as seeking psychological care after these episodes (Figure 1); 19.2% of the participants reported having experienced all symptoms, with no differences between sexes (p=0.550).

**Figure 2:**
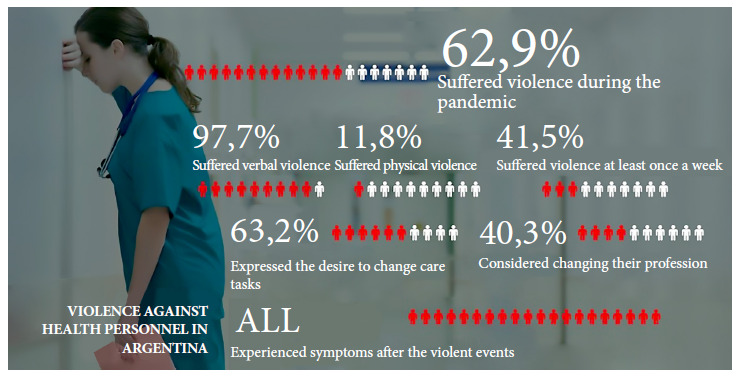
Graphical summary: frequency and impact of acts of violence among healthcare workers in Argentina.

Among the victims of violence, 63.2% acknowledged having considered changing their care work after these episodes, while 40.3% of those assaulted considered abandoning their profession (Figure 2).

In comparison with the rest of Latin America, participants from Argentina reported having experienced violence more frequently (62.9% vs. 54.6%, p<0.001), being significantly more frequent and stressful. They also reported more frequent aggressions against other members of the healthcare team (Table 2).

The reporting rate of violence was low overall, showing a non-significant trend towards underreporting among health workers in Argentina (22.0% vs. 24.4%, p=0.183). Health care workers in Argentina who suffered violence more frequently considered the possibility of changing their care tasks (63.2% vs. 48.4%, p<0.001) and/or desires to change their profession (40.3% vs. 23.6%, p<0.001). Differences in considering changing the profession were due to the opinion of physicians (43.0% among Argentine physicians vs. 23.2%, p<0.001) and nurses (36.8% vs. 26.0%, p=0.045), with no differences among the other groups of workers (p≥0.25).

A Poisson regression was performed to establish whether health personnel in Argentina had experienced more violence than their peers in other Latin American countries. In the crude model, participants from Argentina had a violence prevalence ratio of 1.15 (95% confidence interval [CI]: 1.09-1.22; p<0.001); in the final model (adjusted for all significant covariates), the observed relationship maintained its statistical significance, with a prevalence ratio value of 1.15 (95% CI: 1.09-1.21; p<0.001) (Table 3). Other characteristics associated with higher prevalence of violence episodes were: being a woman, being under 50 years of age, being a physician, nurse, administrative staff, working with patients with COVID-19, and working in the public sector (Table 3).

**Table 3:** Variables associated with suffering violence of any type by health personnel.

Variable	Violence against health personnel			
	Crude model		Adjusted model	
	PR (95% CI)	p-value	aPR (95% CI)	p-value
Country of origin				
Other countries	Ref.		Ref.	
Argentina	1.15 (1.09-1.22)	<0.001	1.15 (1.09-1.21)	<0.001
Sex				
Men	Ref.		Ref.	
Women	1.29 (1.21-1.36)	<0.001	1.19 (1.13-1.25)	<0.001
Age				
>50 years	Ref.		Ref.	
36 to 50 years	1.58 (1.44-1.73)	<0.001	1.38 (1.27-1.50)	<0.001
≤35 years	1.79 (1.63-1.96)	<0.001	1.57 (1.44-1.70)	<0.001
Works with COVID-19 patients				
No	Ref		Ref.	
Yes	1.86 (1.70-2.04)	<0.001	1.65 (1.52-1.80)	<0.001
Role				
Other ^a^	Ref.		Ref.	
Physicians	1.17 (1.03-1.33)	0.016	1.22 (1.08-1.36)	0.001
Nurses	1.37 (1.12-1.57)	<0.001	1.20 (1.06-1.36)	0.004
Kinesiologists	0.84 (0.66-1.07)	0.158	0.74 (0.59-0.93)	0.008
Administrative	1.42 (1.18-1.70)	<0.001	1.42 (1.21-1.68)	<0.001
Field of work				
Private sector	Ref.		Ref.	
Public sector	1.35 (1.26-1.45)	<0.001	1.18 (1.11-1.26)	<0.001

PR, crude prevalence ratio; aPR, adjusted prevalence ratio; 95% CI, 95% confidence Interval; Ref, reference category.

a Technicians in different areas, biochemists, nutritionists, speech therapists.

Finally, the presence of collinearity between the model variables was evaluated by calculating the VIF. The VIF value for residing in Argentina was 1.04, while the mean of the values of all the variables in the model was 1.71. All the VIF values of the variables included in the final model were less than 3.80 (Supplementary Material).

## DISCUSSION

The main findings of our study are: i) a high proportion of participants reported having been victims of violence during the COVID-19 pandemic; ii) victims of violence experienced a high frequency of cognitive and psychosomatic symptoms, while a significant proportion of them considered changing their job or profession; iii) less than one in four victims of violence reported violence; and iv) participants from Argentina experienced more violence than respondents from other countries, regardless of other personal or demographic characteristics considered.

The problem of violence against health personnel in the context of the COVID-19 pandemic has been previously reported by several authors. In our region, a study conducted in Brazil included 1166 health professionals, most of the participants being women (75%); 54.9% were physicians ^16^. According to the authors, 47.6% of the participants experienced some type of violence. Similar to our findings, younger people and those who cared for patients with COVID-19 were more likely to suffer violent acts ^16^. Another survey conducted in Peru, which included 200 physicians caring for patients infected with COVID-19, reported that 84.5% of the participants experienced at least one act of violence, mostly in women ^17^.

Although it might be assumed that the problem of violence against health personnel is more frequent in low- and middle-income countries ^18^^-^^22^, several publications have warned that this is a cause for alarm and concern even in developed countries and referral centers ^7^^,^^23^^,^^24^. This is a problem that affects the entire healthcare team ^22^, affecting nurses ^16^^,^^25^^,^^26^ and other members of the health care team ^16^, as shown by our survey. Two recently published meta-analyses ^27^^,^^28^ that included 14 and 17 studies, respectively, reported an overall prevalence of violence against healthcare workers during the COVID-19 pandemic ranging from 42% to 47%. Ramzi *et al*. ^27^ evaluated the differences in the prevalence of violence according to the continents where the studies were conducted; the region of the Americas presented a prevalence of 58%, a figure very similar to our findings.

However, perhaps more worrisome than the exact prevalence of these events are the repercussions they have on those who suffer them. Thus, all the participants who were victims of some type of violence reported having suffered different kinds of symptoms after these events. In line with our findings, other authors have also reported that healthcare workers who are victims of violence frequently suffer psychological symptoms and sequelae. Among the main consequences described are post-traumatic stress, psychological distress, and even the development of a marked lack of empathy with patients ^29^^,^^30^. It is disturbing that one out of two to three participants in our study considered changing care tasks or wished to change their profession because of it.

Our results should be interpreted with caution, particularly those regarding the differences in the frequency and characteristics of the aggressions suffered by the participants from Argentina compared to the rest of the participants from Latin America. Although it is possible that differences in the idiosyncrasies of each country may have played a role, it cannot be ruled out that the differences are due to variations in the number of participants included from each country. This could have been favored by the fact that the principal investigators of the project resided in that country. Thus, other studies conducted in the region prior to the onset of the COVID-19 pandemic also found very high rates of violence against physicians and nurses ^31^^-^^36^. However, all of these studies were conducted in a single city or metropolitan area, with the exception of one ^36^. Thus, the only study to date that evaluated the occurrence of violence against health personnel in different Latin American countries used participants from Argentina as a benchmark for comparison with their peers in other countries ^36^. Therefore, we do not have evidence to explain the differences detected, beyond hypothetical possibilities.

Some of our findings raise important questions. It is striking that less than one in four victims of violence reported having made any kind of report of these acts. No publications have been found that allow us to infer the reason for this behavior. In fact, a Spanish study that evaluated the occurrence of violence against healthcare professionals in the region of Madrid based its data on the reports made by them ^37^. Thus, the authors of that study acknowledged that the way in which the data were obtained could have led to an underestimation of the real frequency of these episodes. Other intriguing findings are the gender differences found in the victims of violence, and the low rate of seeking psychological help, despite the presence of cognitive and psychosomatic symptoms. Such aspects have not been addressed by previous studies ^31^^-^^38^, and further studies will be required to confirm these observations, as well as to explain them.

In view of the above, we consider it necessary for future studies to evaluate more exhaustively the differences in episodes of violence against health personnel in the continent, with emphasis on an equitable sampling of the countries of the region. In addition, other types of studies, such as qualitative research, should be conducted to answer some of the issues raised by the present survey, such as the lack of reporting by health personnel who are victims of violence, or gender differences in the occurrence of these acts. Future studies should explore the differences in these episodes during and after the COVID-19 pandemic, and/or during episodes of high and low demand for care. Finally, we believe it is necessary to develop public policies in Latin America to prevent acts of violence against health personnel, facilitating reporting when they occur. In addition, actions should be developed to provide assistance and support to the victims of these acts, with the aim of mitigating their negative repercussions.

Our study has some limitations that should be taken into account when interpreting the results. First, the survey used was developed for this study and has not been validated. However, it should be kept in mind that there is no validated questionnaire in Spanish that addresses the problem of violence against health personnel. Furthermore, due to the level of education and age of most of the participants, there is no reason to consider that the responses were biased due to difficulties in understanding the questions. Besides, we used non-probability sampling by convenience, which carries a risk of selection and reporting bias. Thus, it is possible that people who have experienced violence may be more likely to participate in this survey. Similarly, non-medical health personnel are underrepresented. Since no external data set was available to validate the model, its predictive capacity could be overestimated. In addition, the virtual nature of the survey is *per se* a limitation of the study, since, despite the different strategies, it is not possible to completely eliminate the risk that the survey could have been completed by professionals who were not involved in healthcare activities during the pandemic. In spite of this, we should point out that our study is pioneering in including these non-medical members of the healthcare team, since they have not usually been considered in similar studies. Despite the limitations mentioned above, this study has included a large number of participants from Argentina and the region to assess the prevalence of violence against health care personnel in the context of the COVID-19 pandemic.

In conclusion, our findings suggest that there was a high prevalence of violence against health personnel in Argentina during the COVID-19 pandemic. These events had a high negative impact on those who suffered them; thus, all study participants who suffered violence reported having experienced some psychosomatic symptom after the event. In addition, almost half of the health personnel who experienced violence during the pandemic acknowledged that they had considered changing their profession because of it. Despite this, it was noted that the reporting rate of these events was very low. Violence against health personnel should be included as a priority issue for the institutions and governments of the region. Actions should be implemented to prevent assaults, but at the same time strategies should be developed to detect these acts early, facilitate the process of reporting and seeking help, as well as establish strategies to mitigate their impact on the individuals who suffer them.
