# Supplementary material for: Comparison of violence and aggressions suffered by health personnel during the COVID-19 pandemic in Argentina and the rest of Latin America
Source: Rev Peru Med Exp Salud Publica. 2023 Jun 30;40(2):179–88. doi: 10.17843/rpmesp.2023.402.12646 (PMC10953667; doi:10.17843/rpmesp.2023.402.12646)
Supplement: Supplementary material. — Available in the electronic version of the RPMESP. [file rpmesp-40-02-12646-s001.docx]

**MATERIAL SUPLEMENTARIO**

**Comparación de la violencia y agresiones sufridas por el personal de salud durante la pandemia de COVID-19 en Argentina y el resto de Latinoamérica.**

| **ÍNDICE** | Pág |
| --- | --- |
| Encuesta utilizada durante el estudio | 2 |
| Tabla S1: Definiciones de violencia (basadas en las guías de la Organización Mundial de la Salud) | 6 |
| Tabla S2: Distribución de los participantes por países  Tabla S3: Factor de Inflación de la Varianza (VIF) del modelo final | 7  8 |
| Checklist for Reporting Of Survey Studies (CROSS) | 9 |
|  |  |

**ENCUESTA UTILIZADA DURANTE EL ESTUDIO:**

- Género
  - Hombre, Mujer, Prefiero no decirlo
- Edad
- País de residencia
- ¿Cuál es su rol en el sistema de salud?
  - Administrativa/o
  - Bioquímica/o y personal de laboratorio
  - Camillera/o
  - Enfermera/a
  - Farmacéutica/o
  - Kinesiologa/o
  - Médica/o
  - Secretaria/o
  - Técnica/o radióloga/0
  - Otros (telefonistas, instrumentador/a quirúrgica/o, auxiliares, etc)
- En caso de ser médica/o, posee alguna especialidad
  - Sí, No, Prefiero no decirlo
- Especialidad
- Años desde que se recibió
  - Menor de 5 años
  - 5 a 10 años
  - 11 a 15 años
  - Más de 15 años
  - Prefiero no decirlo
- Ámbito de trabajo
  - Público
  - Privado
  - Público y privado
  - Prefiero no decirlo
- ¿Trabaja usted regularmente con pacientes con COVID-19?
  - Sí, No, No estoy seguro
- ¿Has sufrido algún hecho de violencia entre Marzo de 2020 hasta el presente? RECUERDE que la encuesta se focaliza en violencia recibida de parte de PACIENTES/FAMILIARES; quedan excluidos los hechos de violencia sufridos por parte de colegas, otros miembros de salud o personal del ámbito laboral
  - Sí, No, No estoy seguro
- Detalles del evento de violencia: Si ha vivido y desea relatar más de un evento de violencia, tendrá en uno minutos la oportunidad de hacerlo. En dicho caso le pedimos que detalle aquí su vivencia respecto al evento de violencia más relevante para Ud.
  - ¿Sufrió violencia física?
    - Sí, No, No estoy seguro.
  - ¿Sufrió violencia verbal: gritos, insultos, improperios, etc?
    - Sí, No, No estoy seguro.
  - ¿Sufrió otro tipo de violencia?
    - Sí, No, No estoy seguro.
    - ¿Con qué frecuencia ha sufrido los hechos de violencia?Cotidianamente (varias veces en la semana)
    - Muy frecuentemente (aproximadamente una vez por semana)
    - Esporádicamente (pocas veces en el mes)
    - Excepcionalmente
  - ¿En qué ámbito ha sufrido los eventos de violencia?
    - Hospital público
    - Hospital o Institución privada
    - Unidad de pacientes febriles
    - Unidad de cuidados críticos (Terapia intensiva o Unidad Coronaria)
    - Sala de internación general
    - Sala de internación de COVID
  - Por parte de quién ha sufrido violencia
    - Paciente
    - Familiar de paciente
    - Paciente y familiares
    - Paciente, familiares y terceros
    - Otro
  - ¿Cuál fue la actitud de otras personas al presenciar el episodio de violencia?
    - Intercedieron en favor de Ud
    - Se mostraron indiferentes
    - Se sumaron a la agresión
    - No sabría precisarlo
    - No había otras personas presentes
  - ¿Cuál fue la actitud de otros miembros del equipo de salud al presenciar el episodio de violencia?
    - No había otras personas presentes
    - Intercedieron en favor de Ud
    - Buscaron ayuda de terceros
    - Se mostraron indiferentes
    - No sabría precisarlo
  - ¿Cuán estresante diría que ha sido esta experiencia adversa para Ud?
    - 1: No estresante
    - 10: El evento laboral más estresante
  - ¿Luego del evento volvió a experimentar los síntomas (reviviscencia)? La pregunta se refiere a recordar el trauma y sentir ese miedo de nuevo. Ejemplos incluyen: Flashbacks (le hacen sentir como si estuviera pasando por el evento nuevamente), Pesadillas o Pensamientos aterradores.
    - Sí, No, No estoy seguro.
  - ¿Luego del evento padeció síntomas de evasión? Intentó evitar situaciones o personas que desencadenan recuerdos del evento traumático. Esto generó que usted evite lugares, eventos u objetos que le recuerden la experiencia traumática; o evite pensamientos o sentimientos relacionados con el evento traumático. Por ejemplo, puede intentar mantenerse muy ocupado para evitar pensar en lo que sucedió
    - Sí, No, No estoy seguro.
  - Luego del evento padeció síntomas de hipervigilancia y reactividad? Es decir, sentir nerviosismo o estar atento al peligro. Incluyen: sentirse fácilmente sobresaltado; sentirse tenso o "al límite"; tener dificultad para dormir; tener arrebatos de ira
    - Sí, No, No estoy seguro
  - Luego del evento padeció síntomas cognitivos y del estado de ánimo? Consisten en cambios negativos en creencias y sentimientos. Incluyen: problemas para recordar cosas importantes sobre el evento traumático; pensamientos negativos sobre usted o el mundo; sentir culpa y remordimiento; perder interés en cosas que antes disfrutaba; problemas para concentrarse
    - Sí, No, No estoy seguro
  - ¿Debió solicitar atención psicológica luego de esta experiencia?
    - Sí, No, No estoy seguro
  - ¿Pensó en modificar sus actividades asistenciales luego de este episodio de violencia?
    - Sí, No, No estoy seguro
  - ¿Pensó en abandonar la profesión luego de este episodio de violencia?
    - Sí, No, No estoy seguro
  - ¿Realizó algún tipo de denuncia luego de este episodio de violencia?
    - Sí, No
  - ¿Qué tipo de denuncia realizó?
    - No realicé ninguna denuncia
    - Denuncia al jefe de su área
    - Denuncia ante las autoridades de la institución
    - Denuncia policial
    - Denuncia judicial
  - ¿Padeció y/o desea relatar más de un evento de violencia?
    - Sí, No
- Preguntas finales:
  - ¿Ha presenciado hechos de violencia por parte de pacientes o familiares contra algún otro miembro del equipo de Salud desde Marzo de 2020 y hasta la actualidad?
    - Sí, No, No estoy seguro
  - ¿Recuerda haber sufrido violencia por parte de pacientes o familiares fuera del contexto de la pandemia por COVID-19?
    - Sí, No, No estoy seguro

Comentarios o aclaraciones: Cualquier información que crea relevante y que no ha sido preguntada previamente pueda detallarla aquí. Adicionalmente, si desea reportar más de un hecho de violencia y no posee tiempo para completar nuevamente la encuesta, puede colocar los detalles aquí.

**Tabla S1:** Definiciones de violencia (basadas en las guías de la Organización Mundial de la Salud)

| **Término** | **Definición operacional** |
| --- | --- |
| **Violencia en el trabajo** | Cualquier tipo de incidente relacionado con un paciente, familiares de pacientes o cualquier otro individuo que no forme parte de la institución sanitaria, en el cual el personal de salud sienta en riesgo su seguridad, bienestar o salud, de forma explícita o implícita. |
| **Violencia verbal** | Uso intencional de insultos, gritos u otras palabras descalificantes con la finalidad de causar daño o dolor a la persona que los recibe. |
| **Violencia física** | El uso de fuerza física contra otra persona que resulte en daño físico o psicológico. |
| **Otros tipos de violencia** | Se incluye cualquier tipo de daño no contemplado en las definiciones anteriores, así como amenazas, chantaje o extorsión. |
| **Reviviscencia** | Implica volver a experimentar los síntomas, recordar el trauma y volver a sentir ese miedo. Esto incluye: flashbacks (te hace sentir como si estuvieras viviendo el evento nuevamente), pesadillas o pensamientos aterradores. |
| **Síntomas de abstinencia** | Eludir situaciones o personas que evocan recuerdos del evento traumático. Esto genera que evites lugares, situaciones u objetos que te recuerden la experiencia traumática; u obviar pensamientos o sentimientos relacionados con el evento. Por ejemplo, mantenerte ocupado para evitar pensar en lo sucedido. |
| **Hipervigilancia y reactividad** | Sentirse nervioso o estar alerta, en lugares o situaciones en las que no debieras sentirte de esa forma. Esto incluye: asustarse fácilmente, sentirse tenso o “al límite”; sufrir problemas para conciliar el sueño; tener arrebatos de ira. |
| **Síntomas cognitivos o alteraciones del estado de ánimo** | Implica cambios negativos en creencias o sentimientos. Esto incluye: dificultad para recordar hitos importantes respecto al evento traumático; pensamientos negativos sobre uno mismo o el mundo; sentimientos de culpa y remordimiento; pérdida del interés en cosas que antes disfrutaba; problemas para concentrarse. |

**Tabla S2:** País de los participantes:

| **País** | **Porcentaje [“n”]** |
| --- | --- |
| Argentina | 56.30% [1992] |
| Ecuador | 10.85% [384] |
| México | 7.43% [263] |
| Colombia | 4.72% [167] |
| Uruguay | 3.73% [132] |
| Perú | 3.73% [132] |
| Chile | 3.59% [127] |
| Paraguay | 3.48% [123] |
| República Dominicana | 1.53% [54] |
| Guatemala | 1.33% [47] |
| Costa Rica | 1.22% [43] |
| Venezuela | 0.96% [34] |
| Bolivia | 0.45% [16] |
| Nicaragua | 0.37% [13] |
| El Salvador | 0.14% [5] |
| Cuba | 0.08% [3] |
| Honduras | 0.03% [1] |
| Panamá | 0.03% [1] |
| Puerto Rico | 0.03% [1] |

**Tabla S3**: Factor de Inflación de la Varianza (VIF) del modelo final

| Variable | VIF | 1/VIF |
| --- | --- | --- |
| Trabajar en Argentina | 1.04 | 0.957667 |
| Mujeres | 1.08 | 0.928788 |
| Edad  - de 36 a 50 años  - menor a 35 años | 1.71  1.76 | 0.586189  0.569788 |
| Atender pacientes con COVID-19 | 1.09 | 0.916587 |
| Médicos | 3.79 | 0.263825 |
| Enfermeros | 3.13 | 0.319832 |
| Kinesiólogos | 1.44 | 0.693639 |
| Administrativos | 1.53 | 0.655337 |
| Trabajar en el sector público | 1.06 | 0.942607 |

**Checklist for Reporting Of Survey Studies (CROSS)**

| **Section/topic** | **Item** | **Item description** | **Reported on page #** |
| --- | --- | --- | --- |
| **Title and abstract** | | |  |
| Title and abstract | 1a | State the word “survey” along with a commonly used term in title or abstract to introduce the study’s design. | Pág 1 |
|  | 1b | Provide an informative summary in the abstract, covering background, objectives, methods, findings/results, interpretation/discussion, and conclusions. |  |
| **Introduction** | | |  |
| Background | 2 | Provide a background about the rationale of study, what has been previously done, and why this survey is needed. | Pág 3 |
| Purpose/aim | 3 | Identify specific purposes, aims, goals, or objectives of the study. |  |
| **Methods** | | |  |
| Study design | 4 | Specify the study design in the methods section with a commonly used term (e.g., cross-sectional or longitudinal). | Pág 3 |
|  | 5a | Describe the questionnaire (e.g., number of sections, number of questions, number and names of instruments used). | Pág 3 |
| Data collection methods | 5b | Describe all questionnaire instruments that were used in the survey to measure particular concepts. Report target population, reported validity and reliability information, scoring/classification procedure, and reference links (if any). |  |
|  | 5c | Provide information on pretesting of the questionnaire, if performed (in the article or in an online supplement). Report the method of pretesting, number of times questionnaire was pre-tested, number and demographics of participants used for pretesting, and the level of similarity of demographics between pre-testing participants and sample population. | Pág 3-4 |
|  | 5d | Questionnaire if possible, should be fully provided (in the article, or as appendices or as an online supplement). | Mat. Suplementario |
| Sample characteristics | 6a | Describe the study population (i.e., background, locations, eligibility criteria for participant inclusion in survey, exclusion criteria). | Pág 3-4 |
|  | 6b | Describe the sampling techniques used (e.g., single stage or multistage sampling, simple random sampling, stratified sampling, cluster sampling, convenience sampling). Specify the locations of sample participants whenever clustered sampling was applied. | Pág 4 |
|  | 6c | Provide information on sample size, along with details of sample size calculation. | Pág 4 |
|  | 6d | Describe how representative the sample is of the study population (or target population if possible), particularly for population-based surveys. |  |
| Survey  administration | 7a | Provide information on modes of questionnaire administration, including the type and number of contacts, the location where the survey was conducted (e.g., outpatient room or by use of online tools, such as SurveyMonkey). | Pág 3-4 |
|  | 7b | Provide information of survey’s time frame, such as periods of recruitment, exposure, and follow-up days. | Pág 4 |
|  | 7c | Provide information on the entry process:  –>For non-web-based surveys, provide approaches to minimize human error in data entry.  –>For web-based surveys, provide approaches to prevent “multiple participation” of participants. | No aplica  Pág 4 |
| Study preparation | 8 | Describe any preparation process before conducting the survey (e.g., interviewers’ training process, advertising the survey). | Pág 4 |
| Ethical considerations | 9a | Provide information on ethical approval for the survey if obtained, including informed consent, institutional review board [IRB] approval, Helsinki declaration, and good clinical practice [GCP] declaration (as appropriate). | Pág 4 |
|  | 9b | Provide information about survey anonymity and confidentiality and describe what mechanisms were used to protect unauthorized access. | Pág 4 |
| Statistical  analysis | 10a | Describe statistical methods and analytical approach. Report the statistical software that was used for data analysis. | Pág 4-5 |
|  | 10b | Report any modification of variables used in the analysis, along with reference (if available). | Pág 4-5 |
|  | 10c | Report details about how missing data was handled. Include rate of missing items, missing data mechanism (i.e., missing completely at random [MCAR], missing at random [MAR] or missing not at random [MNAR]) and methods used to deal with missing data (e.g., multiple imputation). | Pag 4 |
|  | 10d | State how non-response error was addressed. | Pág 4 |
|  | 10e | For longitudinal surveys, state how loss to follow-up was addressed. | No aplica |
|  | 10f | Indicate whether any methods such as weighting of items or propensity scores have been used to adjust for non-representativeness of the sample. | No aplica |
|  | 10g | Describe any sensitivity analysis conducted. | Pág 4 |
| **Results** | | |  |
| Respondent characteristics | 11a | Report numbers of individuals at each stage of the study. Consider using a flow diagram, if possible. | Pág 5 |
|  | 11b | Provide reasons for non-participation at each stage, if possible. | No aplica |
|  | 11c | Report response rate, present the definition of response rate or the formula used to calculate response rate. | No aplica |
|  | 11d | Provide information to define how unique visitors are determined. Report number of unique visitors along with relevant proportions (e.g., view proportion, participation proportion, completion proportion). | No aplica |
| Descriptive  results | 12 | Provide characteristics of study participants, as well as information on potential confounders and assessed outcomes. | Pág 5 |
| Main findings | 13a | Give unadjusted estimates and, if applicable, confounder-adjusted estimates along with 95% confidence intervals and p-values. | Pág 5-6 |
|  | 13b | For multivariable analysis, provide information on the model building process, model fit statistics, and model assumptions (as appropriate). | Pág 4-6 y Mat. Suplementario |
|  | 13c | Provide details about any sensitivity analysis performed. If there are considerable amount of missing data, report sensitivity analyses comparing the results of complete cases with that of the imputed dataset (if possible). | No aplica |
| **Discussion** | | |  |
| Limitations | 14 | Discuss the limitations of the study, considering sources of potential biases and imprecisions, such as non-representativeness of sample, study design, important uncontrolled confounders. | Pág 7 |
| Interpretations | 15 | Give a cautious overall interpretation of results, based on potential biases and imprecisions and suggest areas for future research. | Pág 8 |
| Generalizability | 16 | Discuss the external validity of the results. | Pág 6-7 |
| **Other sections** | | |  |
| Role of funding source | 17 | State whether any funding organization has had any roles in the survey’s design, implementation, and analysis. | Pag 2, primer documento |
| Conflict of interest | 18 | Declare any potential conflict of interest. | Pag 2, primer documento |
| Acknowledgements | 19 | Provide names of organizations/persons that are acknowledged along with their contribution to the research. | Pag 8 |
