# Supplementary material for: Comparison of violence and aggressions suffered by health personnel during the COVID-19 pandemic in Argentina and the rest of Latin America
Source: Rev Peru Med Exp Salud Publica. 2023 Jun 30;40(2):179–88. doi: 10.17843/rpmesp.2023.402.12646 (PMC10953667; doi:10.17843/rpmesp.2023.402.12646)
Supplement: Translation

# Translation: Comparación de la violencia y agresiones sufridas por el personal de salud durante la pandemia de COVID-19 en Argentina y el resto de Latinoamérica

**Authors:** Sebastián Garcia-Zamora, Pablo A. Iomini, Laura Pulido, Andrés F. Miranda-Arboleda, Pilar Lopez-Santi, Lucrecia M. Burgos, Gonzalo E. Perez, Mauricio Priotti, Darío E. García, Melisa Antoniolli, Gabriel Musso, Ezequiel J. Zaidel, Álvaro Sosa-Liprandi, Mildren A. Del-Sueldo, Ricardo Lopez-Santi, Gustavo Vazquez, Adrián Baranchuk

**Affiliations:** 1 Servicio de Cardiología, Sanatorio Delta, Rosario, Argentina.; 2 Facultad de Medicina, Universidad de Buenos Aires (UBA) - UDH Hospital Dr. Prof. Alejandro Posadas, Buenos Aires, Argentina.; 3 Servicio de Neumonología, Sanatorio Centro, Rosario, Argentina.; 4 División de Cardiología, Queen’s University, Kingston, Ontario, Canadá.; 5 División de Cardiología, Hospital Italiano de La Plata, La Plata, Argentina.; 6 Instituto Cardiovascular de Buenos Aires, Argentina.; 7 División de Cardiología, Clínica Olivos, Buenos Aires, Argentina.; 8 Federacion Latinoamericana de Medicina de Emergecias, Ciudad de México, México; 9 Servicio de Cardiología, Sanatorio Finochietto, Buenos Aires, Argentina.; 10 Servicio de Terapia Intensiva, Sanatorio Parque, Rosario, Argentina.; 11 Departamento de Cardiología, Sanatorio Güemes, CABA, Argentina.; 12 Fundación Certus-Clínica de Especialidades, Córdoba, Argentina.; 13 Departamento de Psiquiatría, Queen’s University, Kingston, Ontario, Canadá.

**Keywords:** Violencia, COVID-19, Pandemia, Agresión, América Latina, Personal de Salud

## Abstract

**Objetivos.:**

Explorar la frecuencia e impacto de la violencia contra los trabajadores de salud de Argentina y compararlo con el resto de sus pares de Latinoamérica en el contexto de la pandemia por COVID-19.

**Materiales y métodos.:**

Estudio de corte transversal a través de una encuesta electrónica al personal médico y no médico de Latinoamérica que desempeño tareas asistenciales desde marzo de 2020. Se utilizó una regresión de Poisson para estimar las Razones de Prevalencia crudas (RP) y ajustadas (RPa) con sus respectivos intervalos de confianza al 95%.

**Resultados.:**

Un total de 3544 participantes de 19 países respondieron la encuesta; 1992 (56,0%) residían en Argentina. Entre los mismos, el 62,9% padeció al menos un hecho de violencia: 97,7% refirió violencia verbal y 11,8% violencia física. El 41,5% de los agredidos padecieron situaciones de violencia al menos una vez por semana. El personal de salud de Argentina vivenció más frecuentemente violencia que los de otros países (62,9% *vs*. 54,6%, p<0,001), siendo estos eventos más habituales y estresantes (p<0,05). Además, refirieron más frecuentemente haber considerado cambiar sus tareas asistenciales y/o deseos de abandonar su profesión (p<0,001). En la regresión de Poisson, los participantes de Argentina tuvieron una prevalencia de violencia mayor que los trabajadores de salud de la región (14,6%; p<0,001).

**Conclusiones.:**

Existió una elevada prevalencia de violencia contra el personal de salud de Argentina durante la pandemia por COVID-19. Estos hechos tuvieron un alto impacto negativo entre quienes los padecieron. Nuestros datos sugieren que, esto podría haber sido más frecuente en Argentina respecto de otras regiones del continente.

## INTRODUCCIÓN

La pandemia por COVID-19 ha provocado múltiples repercusiones negativas tanto en los individuos como en los sistemas de salud ^1^^-^^3^. El distanciamiento social, los problemas económicos y laborales, y los cambios de hábitos y rutinas de los individuos durante este período han sido vinculados a un mayor grado de estrés, ansiedad y depresión en distintas sociedades ^2^. Paralelamente, el personal de salud se ha visto sobrecargado de actividades asistenciales, debiendo modificar sus formas de trabajo, y alterando sus rutinas para ayudar a sobrellevar esta compleja situación ^4^. En este escenario desfavorable, un problema que se ha suscitado son las agresiones contra el personal de salud ^5^^-^^7^.

La violencia contra el personal de salud es un fenómeno global, el cual preocupa desde hace décadas ^8^^-^^11^. En el año 2002, la Organización Mundial de la Salud (OMS) y organizaciones asociadas delinearon las directrices para abordar esta problemática. En ellas, definieron a la violencia en el lugar de trabajo como «incidentes en los que el personal es abusado, amenazado o agredido en circunstancias relacionadas con su trabajo […] las cuales implican un desafío explícito o implícito a su seguridad, bienestar o la salud» ^12^.

A pesar de lo antes mencionado, poco se conoce respecto a lo que ha ocurrido en Latinoamérica o Argentina luego del inicio de la pandemia por COVID-19. Debido a ello, la Sociedad Interamericana de Cardiología (SIAC) llevó adelante una encuesta para explorar la frecuencia y características de los hechos de violencia contra trabajadores de la salud de América Latina durante la pandemia por COVID-19 ^13^. En esta encuesta, más del 50% de los participantes manifestaron haber padecido algún episodio de violencia laboral durante la pandemia. A pesar de lo elevado de dicha cifra, este fenómeno no parece privativo de Latinoamérica; así, estudios similares en China y Estados Unidos han reportado cifras superiores al 65%, llegando incluso al 80% de los participantes de algunos estudios ^6^^,^^7^. El impacto de las variaciones regionales en la frecuencia de hechos de violencia de la encuesta de SIAC no está claro. En el presente subestudio, se propuso explorar la frecuencia de los hechos de violencia, y las características e impacto de estos eventos sobre los trabajadores de la salud de Argentina, por tratarse del país con mayor número de participantes del estudio, y compararlo con el resto de sus pares de Latinoamérica en el contexto de la pandemia por COVID-19.

**MENSAJES CLAVE**

Motivación para realizar el estudio. La pandemia por COVID-19 ha generado profundas repercusiones en diferentes niveles socio-ambientales. Su impacto sobre la violencia contra los miembros del equipo de salud de Argentina no ha sido bien documentado.

Principales hallazgos. El presente estudio evidenció altas tasas de agresiones, especialmente de tipo verbal. Además, casi la mitad de los participantes relató haber sufrido estos hechos con una periodicidad semanal. Todos los participantes que padecieron violencia manifestaron haber experimentado síntomas posteriores a dicho evento; y hasta un tercio manifestó haber considerado abandonar su profesión luego de estos actos.

Implicancias. Resulta imperioso realizar acciones para prevenir los actos de violencia contra el personal de salud, o mitigar su impacto entre las víctimas de los mismos.

## MATERIALES Y MÉTODOS

### Diseño y población de estudio

El desarrollo e implementación del proyecto han sido publicados previamente ^13^. En resumen, se implementó un estudio de tipo corte transversal mediante una encuesta electrónica autoadministrada, que fue diseñada específicamente para este estudio. La encuesta se aplicó desde el 11 de enero y el 28 de febrero de 2022, invitando a participar de la misma a todo el personal de salud médico y no médico de América Latina. La participación en la encuesta fue voluntaria y anónima, y esta información se proporcionó a los participantes al inicio del cuestionario. Se definió como personal de salud a todos los miembros del sistema, incluyendo médicos, enfermeros, kinesiólogos, bioquímicos, técnicos en distintas áreas (diagnóstico por imágenes, prácticas cardiológicas, laboratorio, entre los principales), y personal administrativo que tuviera contacto con pacientes o familiares. Respecto a los médicos, no se realizaron restricciones por especialidad o trabajo realizado, lugar de residencia, edad u otras variables. Se excluyeron del presente estudio a aquellos individuos que no realizaban actividades asistenciales desde el inicio de la pandemia en la región, fijando como límite temporal para ello el mes de marzo de 2020. Por no disponer de datos fidedignos respecto al número de profesionales de la salud de Latinoamérica, no se realizó un cálculo del tamaño muestral.

El cuestionario fue distribuido de manera electrónica a través de invitaciones enviadas a las casillas de correo electrónico de los miembros de SIAC, tanto por esta sociedad como por medio de las sociedades científicas que la componen. Además, durante el lapso de tiempo que se llevó a cabo la encuesta, se invitó a participar de esta iniciativa a través de las plataformas de redes sociales de SIAC (Twitter, WhatsApp e Instagram).

### Cuestionario

La encuesta fue diseñada en Google Forms (Mountain View, CA), siguiendo las recomendaciones del Consenso para Reportar Estudios de Encuestas (CROSS, por sus siglas en inglés) propuesta por la Red de Mejora de la Calidad y Transparencia de la Investigación en Salud (EQUATOR, por sus siglas en inglés) ^14^. Se incluyeron 49 preguntas divididas en cinco secciones, donde se recabaron datos demográficos de los participantes, formación y experiencia de estos, grado de vinculación con pacientes con infección activa por COVID-19, y preguntas relacionadas con posibles experiencias de violencia o agresiones padecidas durante el período analizado (Material Suplementario).

Debido al dinamismo de los sucesos y repercusiones de la pandemia por COVID-19, y dado que los potenciales participantes eran individuos con un grado de instrucción elevado, se decidió no realizar una prueba piloto del cuestionario.

### Variables y mediciones

Las definiciones de los distintos tipos de violencia se basaron en las directrices de la OMS ^12^^,^^13^. Así, se consideró como hechos de violencia a cualquier tipo de incidente relacionado con pacientes, familiares de pacientes o cualquier otro individuo que no forme parte de la institución sanitaria, donde el personal de salud haya sentido que corría riesgo su seguridad. La definición de otros tipos de violencia, como así también las consecuencias psicológicas de estos hechos (reviviscencia, síntomas de abstinencia, hipervigilancia y reactividad, y síntomas cognitivos o alteraciones del estado de ánimo) se encuentran descritas en detalle en el Material Suplementario.

Para la valoración del nivel de estrés percibido por los participantes que experimentaron eventos de violencia se utilizó una escala lineal tipo Likert. Se solicitó a los participantes que habían sufrido algún acto de violencia que indicaran cuán estresante había sido dicha experiencia para ellos, utilizando una escala del 1 al 10. En esta escala, asignar un puntaje de 1 significaba que el hecho no había sido estresante, mientras que un puntaje de 10 equivalía al evento laboral más estresante que habían experimentado (Material Suplementario).

### Análisis estadístico

Las variables continuas se expresaron como media y desviación estándar o mediana y rango intercuartílico, según su distribución. La normalidad de cada variable se evaluó mediante herramientas gráficas (histogramas y diagramas de probabilidad normal) y la prueba de Shapiro-Wilk. Las variables categóricas se expresaron mediante valores absolutos y porcentajes. Se utilizó la prueba T de Student para las comparaciones entre grupos de variables continuas que se distribuyeron normalmente. Las comparaciones entre proporciones se realizaron mediante la prueba chi-cuadrado o la prueba exacta de Fisher según la frecuencia de los valores esperados. Se detectaron datos faltantes en tres ítems de la encuesta: sexo de los participantes (0,3%), país de residencia (0,2%), y año de recibido (3,3%). Sin embargo, no se aplicó ningún tipo de estrategia para compensar esto.

Posteriormente, se construyó un modelo de regresión para explorar si los participantes de Argentina experimentaron episodios de violencia con una frecuencia diferente al personal de salud de otros países de Latinoamérica. Con el objetivo de estimar la razón de prevalencia de los eventos de violencia entre los participantes, se utilizó una regresión de Poisson. En primer lugar, se calculó la razón de prevalencia de padecer algún tipo de violencia para todas las variables recopiladas en la encuesta (análisis crudo). Luego, se procedió a construir un modelo ajustado de forma manual, incluyendo solo las variables que obtuvieron un valor de p<0,20 en el modelo crudo. En el modelo final ajustado, solo se mantuvieron las variables que mostraron significancia estadística (p<0,05). Finalmente, se exploró la presencia de colinealidad en las variables del modelo ajustado mediante el cálculo del factor de inflación de la varianza (VIF, por sus siglas en inglés). Un VIF de 1 indicó la ausencia de colinealidad, mientras que un valor mayor a 10 se consideró como elevada colinealidad. Se estableció la significación estadística como un valor de p<0,05 en pruebas «a dos colas» para todas las pruebas estadísticas realizadas. Todos los análisis se llevaron a cabo utilizando Stata versión 13.0 (StataCorp, College Station, TX, USA).

### Aspectos éticos

El protocolo de investigación fue aprobado por el comité de Ética de la SIAC, y no se puso en riesgo, bajo ninguna circunstancia, la integridad o la información de los participantes. Con el objetivo de garantizar el anonimato y fomentar la veracidad de las respuestas, no se solicitó a los participantes ningún dato identificativo ni se requirió un consentimiento por escrito. De esta manera, se consideró que quienes respondieron la encuesta otorgaron su consentimiento informado de manera tácita para participar en el estudio ^15^. A fin de garantizar la no duplicación de las respuestas, se programó el cuestionario para recabar obligatoriamente el correo electrónico de los participantes. Únicamente el investigador principal tuvo acceso a estos datos, y se encargó de llevar a cabo la anonimización de los mismos, garantizando así la confidencialidad de los participantes.

## RESULTADOS

Respondieron a la encuesta 3544 participantes de 19 países; de ellos, el 56,2% (n=1992) residían en Argentina. El 63,0% de los participantes de Argentina fueron mujeres, y la edad media de los mismo fue 42,6 ± 10,7 años. El 68,4% eran médicos, 19,9% enfermeros, 3,2% kinesiólogos, 3,0% eran secretarios o administrativos, y el 5,5% restante tenía otras funciones dentro del equipo de salud. El 91,5% de los médicos manifestó ser especialista: 34,4% eran cardiólogos, 10,5% intensivistas o emergentólogos, 11,4% tenían alguna especialidad quirúrgica, 9,4% eran pediatras o tenían alguna subespecialidad afín. El 75,1% de los participantes refirió asistir regularmente a pacientes con COVID-19. En la Tabla 1 se comparan las características basales de los participantes de Argentina y los del resto del continente.

**Tabla 1:** Características basales de los participantes de Argentina comparado con otros países de América.

Característica	Argentina (n=1992)	Resto de Latinoamérica (n=1552)	Valor de p^a^
	n (%)	n (%)	
Sexo (n=3532)			
Mujeres	1251 (63,0)	816 (52,8)	<0,001
Hombres	734 (37,0)	731 (47,2)	
Edad (en años)^b^	42,6 ± 10,7	41,1 ± 11,3	<0,001^c^
Personal de Salud (n=3544)			
Médicos	1363 (68,4)	1147 (73,9)	<0,001
Enfermeros	397 (19,9)	170 (11,0)	<0,001
Kinesiólogos	63 (3,2)	56 (3,6)	0,465
Administrativos	59 (3,0)	41 (2,6)	0,568
Otros ^d^	110 (5,5)	138 (8,9)	<0,001
Especialistas (n=2498)			
Sí	1243 (91,5)	882 (77,4)	<0,001
No	116 (8,5)	257 (22,6)	
Tiempo de práctica (n=3364)			
Hasta 10 años	728 (38,2)	717 (49,1)	<0,001
De 11 a 15 años	383 (20,1)	210 (14,4)	
Más de 15 años	793 (41,7)	533 (36,5)	
Ámbito de trabajo (n=3525)			
Público	677 (34,1)	610 (39,6)	0,001
Privado	614 (31,0)	405 (26,3)	
Público y privado	693 (34,9)	526 (34,1)	
Atención de pacientes con COVID-19 (n=3544)			
Sí	1496 (75,1)	1151 (74,2)	0,809
No	496 (24,9)	401 (25,8)	

a calculado mediante la prueba chi-cuadrado, ^b^ Media ± desviacion estandar; ^c^ calculado mediante la prueba T de *student*; ^d^ técnicos en distintas áreas, bioquimicos, nutricionistas, y fonoaudiólogos.

Entre la población argentina, el 62,9% refirió haber padecido al menos un hecho de violencia luego del inicio de la pandemia por COVID-19. El 97,7% padeció violencia verbal, 11,8% violencia física y 39,4% otros tipos de violencia. Los agredidos calificaron el nivel de estrés de estos hechos con un puntaje promedio de 8,4 ± 1,7 puntos. Si bien no hubo diferencias en la frecuencia de agresiones entre Argentina y otros países según el sexo (97,7% en mujeres *vs*. 97,6% en hombres, p=0,945), las mujeres vivenciaron estos hechos de manera ligeramente más estresantes (asignando un puntaje promedio de 8,6 ±1,5 *vs*. 8,2 ±1,9 en hombres, p<0,001). El 12,9% de los agredidos manifestó que padecía algún tipo de violencia con una frecuencia diaria; un 28,6% vivenciaban esto aproximadamente una vez a la semana, y el 36,8% expresó padecerlo algunas pocas veces al mes. Las agresiones fueron realizadas principalmente por los pacientes y sus familiares (34,7%), o sólo por familiares (31,0%) (Tabla 2).

**Tabla 2:** Diferencias en los patrones de violencia entre el personal de salud de Argentina y el de otras regiones de América Latina.

Características	Argentina (n=1992)	Otras regiones (n=1552)	Valor de p^a^
	n (%)	n (%)	
Violencia global			
Sí	1253 (62,9)	847 (54,6)	<0,001
No	739 (37,1)	705 (45,4)	
Violencia verbal			
Sí	1224 (97,7)	816 (96,3)	0,069
No	29 (2,3)	31 (3,7)	
Violencia física			
Sí	148 (11,8)	85 (10,0)	0,204
No	1105 (88,2)	762 (90,0)	
Otros tipos de violencia			
Sí	493 (39,4)	259 (30,6)	<0,001
No	760 (60,6)	588 (69,4)	
Tiempo de ocurrencia de hechos violentos			
Violencia semanal^b^	520 (41,5)	309 (36,5)	0,021
Violencia menos de 1 vez a la semana	733 (58,5)	538 (63,5)	
Puntaje de estrés percibido^c,d^	8,4 ±1,7	7,8 ±2,1	<0,001^d^
Requirió atención psicológica			
Sí	164 (13,1)	96 (11,3)	0,231
No	1089 (86,9)	751 (88,7)	
Evidenció violencia contra otros miembros del equipo de salud			
Sí	1548 (77,7)	1107 (71,3)	<0,001
No	444 (22,3)	445 (28,7)	

a calculado mediante la prueba chi-cuadrado, ^b^ participantes referian haber sufrido episodios de violencia al menos 1 vez por semana; c media ± desviacion estandar; ^d^ basado en un puntaje del 1 al 10, donde 1 es minimamente estresante y 10 el evento mas estresante que han vivido; ^e^ calculado mediante la prueba T de *student.*

Todos los participantes de Argentina que padecieron violencia manifestaron haber experimentado síntomas posteriores a dicho evento, siendo la hipervigilancia el más frecuente (63,2%). La reviviscencia y los síntomas cognitivos fueron más frecuentes entre las mujeres, así como la búsqueda de atención psicológica luego de estos episodios (Figura 1). El 19,2% de los participantes refirió haber experimentado todos los síntomas, sin diferencias entre sexos (p=0,550). Entre las víctimas de violencia, 63,2% reconoció haber considerado cambiar sus tareas asistenciales luego de estos episodios, mientras que el 40,3% de los agredidos consideró abandonar su profesión (Figura 2).

**Figura 1:** Sintomas experimentados por los participantes de Argentina que padecieron al menos un episodio de agresion o abuso, de acuerdo con el sexo.

**Figura 2:** Resumen grafico: frecuencia y repercusiones de los actos de violencia entre los trabajadores de la salud de Argentina.

En comparación con el resto de América Latina, los participantes de Argentina refirieron más frecuentemente haber padecido hechos de violencia (62,9% *vs.* 54,6%, p<0,001), siendo estas significativamente más frecuentes y estresantes. También manifestaron más frecuentemente haber vivenciado agresiones contra otros miembros del equipo de salud (Tabla 2).

La tasa de denuncia de los hechos de violencia fue globalmente baja, mostrando en el personal de salud de Argentina una tendencia no significativa hacia un menor reporte de los mismos (22,0% *vs.* 24,4%, p=0,183). Los trabajadores de la salud en Argentina que sufrieron violencia consideraron más frecuentemente la posibilidad de cambiar sus tareas asistenciales (63,2% *vs*. 48,4%, p<0,001) y/o deseos de abandonar su profesión (40,3% *vs*. 23,6%, p<0,001). Las diferencias en considerar abandonar la profesión se debieron a la opinión de los médicos (43,0% entre los médicos argentinos *vs*. 23,2%, p<0,001) y enfermeros (36,8% *vs.* 26,0%, p=0,045), sin diferencias entre los demás grupos de trabajadores (p≥0,25).

Se realizó una regresión de Poisson para establecer si el personal de salud de Argentina había padecido más violencia que sus pares de otros países de América Latina. En el modelo crudo, los participantes de Argentina tuvieron una razón de prevalencia de violencia de 1,15 (intervalo de confianza [IC] al 95%: 1,09-1,22; p<0,001); en el modelo final (ajustado por todas las covariables significativas), la relación observada mantuvo su significación estadística, con un valor de la razón de prevalencia de 1,15 (IC 95%: 1,09-1,21; p<0,001) (Tabla 3). Otras características asociadas a más prevalencia de episodios de violencia fueron: ser mujer, tener menos de 50 años, ser médico, enfermero, personal administrativo, trabajar con pacientes con COVID-19, y trabajar en el sector público (Tabla 3).

**Tabla 3:** Variables asociadas a padecer violencia de cualquier tipo por el personal de salud.

Variable	Violencia contra el personal de salud			
	Modelo crudo		Modelo ajustado	
	RP (IC 95%)	Valor de p	RPa (IC 95%)	Valor de p
País de residencia				
Residir en otros países	Ref.		Ref.	
Residir en Argentina	1,15 (1,09-1,22)	<0,001	1,15 (1,09-1,21)	<0,001
Sexo				
Varones	Ref.		Ref.	
Mujeres	1,29 (1,21-1,36)	<0,001	1,19 (1,13-1,25)	<0,001
Edad				
>50 años	Ref.		Ref.	
36 a 50 años	1,58 (1,44-1,73)	<0,001	1,38 (1,27-1,50)	<0,001
≤35 años	1,79 (1,63-1,96)	<0,001	1,57 (1,44-1,70)	<0,001
Atender pacientes con COVID-19				
No	Ref		Ref.	
Sí	1,86 (1,70-2,04)	<0,001	1,65 (1,52-1,80)	<0,001
Rol				
Otros ^a^	Ref.		Ref.	
Médicos	1,17 (1,03-1,33)	0,016	1,22 (1,08-1,36)	0,001
Enfermeros	1,37 (1,12-1,57)	<0,001	1,20 (1,06-1,36)	0,004
Kinesiólogos	0,84 (0,66-1,07)	0,158	0,74 (0,59-0,93)	0,008
Administrativos	1,42 (1,18-1,70)	<0,001	1,42 (1,21-1,68)	<0,001
Lugar de trabajo				
Sector privado	Ref.		Ref.	
Sector público	1,35 (1,26-1,45)	<0,001	1,18 (1,11-1,26)	<0,001

RP: razón de prevalencia cruda; RPa: razón de prevalencia ajustada; IC 95%: intervalo de confianza al 95%; Ref: categoría de referencia.

a Técnicos en distintas áreas, bioquímicos, nutricionistas, fonoaudiólogos.

Finalmente, se evaluó la presencia de colinealidad entre las variables del modelo mediante el cálculo del VIF. El valor del VIF para residir en Argentina fue de 1,04, mientras que la media de los valores de todas las variables del modelo fue de 1,71. Todos los valores del VIF de las variables incluidas en el modelo final fueron inferiores a 3,80 (Material Suplementario).

## DISCUSIÓN

Los principales hallazgos de nuestro estudio son: i) una alta proporción de participantes manifestaron haber sido víctimas de violencia durante la pandemia por COVID-19; ii) las víctimas de violencia experimentaron una elevada frecuencia de síntomas cognitivos y psicosomáticos, al tiempo que una importante proporción de los mismos consideró cambiar su trabajo o profesión; iii) menos de uno de cada cuatro víctimas de violencia realizaron algún tipo de denuncia respecto de la misma; y iv) los participantes de Argentina padecieron más violencia que los encuestados de otros países, independientemente de otras características personales o demográficas consideradas.

El problema de la violencia contra el personal de salud en el contexto de la pandemia por COVID-19 ha sido señalado previamente por numerosos autores. En nuestra región, un estudio realizado en Brasil incluyó 1166 profesionales de la salud, siendo la mayoría de las participantes mujeres (75%); 54,9% eran médicas ^16^. De acuerdo con los autores, el 47,6% de los participantes experimentaron algún tipo de violencia. De forma similar a lo observado en nuestro estudio, las personas más jóvenes y aquellos que se desempeñaban al cuidado de pacientes con COVID-19 fueron más propensos a sufrir actos violentos ^16^. En otra encuesta realizada en Perú, en la cual se incluyeron 200 médicos que atendían a pacientes infectados con COVID-19 ^16^, se encontró que el 84,5% de los participantes experimentaron al menos un hecho de violencia con predominio del género femenino en la población ^17^.

Si bien podría pensarse que el problema de la violencia contra el personal de salud predomina en países de bajos y medianos ingresos ^18^^-^^22^, numerosas publicaciones han alertado que esto constituye un motivo de alarma y preocupación inclusive en países desarrollados y en centros de referencia ^7^^,^^23^^,^^24^. Este es un problema que aflige a todo el equipo de salud ^22^, afectando a enfermeros ^16^^,^^25^^,^^26^ y otros miembros del equipo de salud ^16^, como lo muestra nuestra encuesta. Dos meta-análisis publicados recientemente ^27^^,^^28^ que, incluyeron 14 y 17 estudios, respectivamente; reportaron una prevalencia global de violencia contra el personal de salud durante la pandemia por COVID-19 de 42% a 47%. Ramzi *et al.*^27^^)^ evaluaron las diferencias en la prevalencia de violencia de acuerdo con los continentes donde se realizaron los estudios; la región de las Américas presentó una prevalencia del 58%, cifra muy similar a lo hallado en nuestro trabajo.

Sin embargo, quizá más preocupante que la prevalencia exacta de estos hechos, son las repercusiones que tienen sobre quienes los padecen. Así, todos los participantes que fueron víctimas de algún tipo de violencia manifestaron haber padecido síntomas de diferente índole posteriores a estos eventos. En línea con lo encontrado en nuestro estudio, otros autores también han reportado que los trabajadores de la salud víctimas de violencia padecen frecuentemente síntomas y secuelas psicológicas. Entre las principales consecuencias descritas, se encuentran el estrés postraumático, la presencia de distrés psicológico, e incluso el desarrollo de una marcada falta de empatía con los pacientes ^29^^,^^30^. Resulta inquietante que uno de cada dos a tres participantes del presente estudio haya considerado cambiar de tareas asistenciales o desear abandonar su profesión debido a ello.

En cuanto a las diferencias en la frecuencia y características de las agresiones padecidas por los participantes de Argentina comparado con el resto de los participantes de América Latina, se debe ser cautos al interpretar estos hallazgos. Si bien es posible que hayan influenciado diferencias propias de la idiosincrasia de cada país, no puede descartarse que las discrepancias encontradas obedezcan a diferencias en el número de participantes incluidos de cada país. Esto podría haberse visto favorecido por el hecho de que los investigadores principales del proyecto residían en dicho país. Así, otros estudios realizados en la región previo al inicio de la pandemia por COVID-19 encontraron también tasas muy elevadas de violencia contra médicos y enfermeros ^31^^-^^36^. Sin embargo, todos estos estudios fueron realizados en una única ciudad o área metropolitana, a excepción de uno de ellos ^36^. Así, el único estudio hasta el momento que evaluó la ocurrencia de violencia contra el personal de salud en distintos países de América Latina utilizó a los participantes de Argentina como parámetro de referencia para compararlo con sus pares de otros países ^36^. Por lo tanto, no disponemos de evidencia que permita explicar las diferencias detectadas, más allá del terreno hipotético.

Algunas observaciones del presente estudio plantean importantes interrogantes. Así, resulta llamativo que menos de uno de cada cuatro sujetos víctimas de violencia hayan manifestado haber realizado algún tipo de denuncia de estos actos. No se han encontrado publicaciones que permitan inferir el motivo de este comportamiento. De hecho, un estudio español que evaluó la ocurrencia de eventos de violencia hacia profesionales sanitarios de la región de Madrid basó sus datos en las denuncias realizadas por los mismos ^37^. Así, los autores de dicho trabajo reconocen que la forma de obtención de los datos podría haber llevado a una infravaloración de la frecuencia real de estos episodios. Otros hallazgos intrigantes del presente estudio son las diferencias de género observadas en las víctimas de violencia, y la baja tasa de búsqueda de ayuda psicológica, a pesar de la presencia de síntomas cognitivos y psicosomáticos. Dichos aspectos no han sido abordados en estudios previos ^31^^-^^38^, y se requerirán nuevos estudios para confirmar estas observaciones, así como explicar las mismas.

Por todo lo expuesto, consideramos necesario que futuros estudios evalúen de una manera más exhaustiva las diferencias en los episodios de violencia contra el personal de salud del continente, haciendo énfasis en un muestreo equitativo de los países de la región. Además, para responder algunos aspectos surgidos de la presente encuesta, como la falta de denuncia por parte del personal de salud víctima de violencia respecto de estos actos, o las diferencias de género en la ocurrencia de estos hechos, deberían realizarse otro tipo de estudios, como investigaciones cualitativas. Futuros estudios deberían explorar las diferencias de estos episodios durante y posterior a la pandemia por COVID-19, y/o durante episodios de alta y baja demanda asistencial. Finalmente, creemos necesario que se desarrollen políticas públicas en Latinoamérica para prevenir los hechos de violencia contra el personal de salud, facilitando las denuncias cuando los mismos ocurran. Además, se deben desarrollar acciones para favorecer la asistencia y soporte a las víctimas de estos actos, con el objetivo de mitigar sus repercusiones negativas.

Nuestro estudio posee algunas limitaciones que deben ser tenidas en cuenta al momento de interpretar los resultados. En primer lugar, la encuesta utilizada se desarrolló para el presente estudio, y no ha sido validada. Sin embargo, debe tenerse presente que no existe ningún cuestionario validado en nuestro idioma que aborde el problema de la violencia contra el personal de salud. Además, debido al grado de instrucción y la edad de la mayoría de los participantes, no existen motivos para considerar que las respuestas hayan estado sesgadas debido a dificultades en la comprensión de las preguntas. En segundo lugar, el muestreo utilizado fue no probabilístico por conveniencia, lo cual conlleva riesgo de sesgo de selección y reporte. Así, es posible que las personas que hayan vivenciado hechos de violencia posean mayor afición a participar de la presente encuesta. De forma similar, el personal de salud no médico se encuentra subrepresentado. Tercero, por no disponer de un set de datos externos para validar el modelo, la capacidad predictiva del mismo podría encontrarse sobreestimada. En cuarto lugar, el carácter virtual de la encuesta constituye *per se* una limitación del estudio, ya que, pese a las diferentes estrategias utilizadas, no es posible eliminar completamente el riesgo de que la misma haya sido completada por profesionales que no realizaban actividades asistenciales durante la pandemia. A pesar de ello, debemos señalar que nuestro estudio es pionero al incluir a estos miembros no médicos del equipo de salud, ya que habitualmente los mismos no han sido considerados en estudios similares. Pese a las limitaciones señaladas, este estudio ha incluido un gran número de participantes de Argentina y la región para valorar la prevalencia de violencia contra el personal de salud en el contexto de la pandemia por COVID-19.

En conclusión, los hallazgos del presente estudio sugieren que ha existido una elevada prevalencia de violencia contra el personal de salud de Argentina durante la pandemia por COVID-19. Estos hechos tuvieron un alto impacto negativo entre quienes los padecieron; así, todos los participantes del estudio que sufrieron hechos de violencia manifestaron haber experimentado algún síntoma psicosomático posterior a esto. Además, casi la mitad del personal de salud que padeció hechos de violencia durante la pandemia reconoció haber considerado abandonar su profesión debido a esto. A pesar de ello, se observó que la tasa de denuncias de estos hechos fue muy baja. Se considera imperioso que se incluya a la violencia contra el personal de salud como un tema prioritario para las instituciones y los gobiernos de la región. Se deben implementar acciones para prevenir las agresiones, pero al mismo tiempo deben desarrollarse estrategias para detectar estos hechos tempranamente, facilitar los procesos de denuncia y búsqueda de ayuda, y establecer estrategias para mitigar su impacto en los individuos que lo padecen.
